# Changes in Cancer Functional Assessment Set and Functional Independence Measure After Intradural Extramedullary Tumor Resection: A Case Report

**DOI:** 10.7759/cureus.11184

**Published:** 2020-10-26

**Authors:** Junko Nakamura

**Affiliations:** 1 Rehabilitation, Itami Seifu Hospital, Itami, JPN

**Keywords:** intradural extramedullary tumor, functional independence measure, cancer functional assessment set, posterior column of spinal cord

## Abstract

Background and Purpose: The Cancer Functional Assessment Set (cFAS) was developed as a scale for assessing physical function specifically for cancer patients. It is a scale that allows for accurate assessment of physical functioning and the effectiveness of rehabilitation interventions in cancer patients. Here we reported a case of the use of cFAS in rehabilitation after thoracic intradural extramedullary tumor resection.

Case Description: A thoracic spine magnetic resonance imaging scan showed an intradural extramedullary tumor of Th6, which was resected. Postoperatively, the patient was transferred to the recovery unit of our hospital. The residual ataxia symptoms derived from posterior cord symptoms were characteristic of this case.

Intervention: Using cFAS and Functional Independence Measure (FIM) as indices for evaluation, physical therapy was aimed at improving ataxic gait and occupational therapy was performed for impairment of activities of daily living. These treatments were performed for three hours a day during the hospitalization.

Outcomes: Ataxic gait from position sense disturbance due to posterior compression of the spinal cord was present. The FIM (motor component) and cFAS at admission were 58 and 55 points, respectively. Rehabilitation was continued, and the FIM (motor component) and cFAS at discharge were 88 and 86 points, respectively, both showing improvement relative to admission. The patient was discharged 69 days after surgery.

Discussion: Both cFAS and FIM improved after surgery with rehabilitation, reflecting improvement in activities of daily living during recovery from position sense disturbance. Previous cFAS studies had included gastrointestinal, pulmonary, brain, hematologic, genitourinary, genitourinary, head and neck cancers. cFAS may furthermore be a useful tool for assessing spinal cord tumors, particularly in the presence of posterior cord injury.

## Introduction

The relationship between the level of spinal cord injury and the concurrent appearance of symptoms is important in the rehabilitation of spinal cord tumors. Although the American Spinal Injury Association (ASIA) classification has been presented as an assessment derived from spinal cord injury, there was no index of activities of daily living derived from posterior cord injury to the spinal cord [1-2]. When using the Cancer Functional Assessment Set (cFAS) [3], a measure of activities of daily living in cancer proposed by Miyata et al. in 2014, an accurate assessment may be possible because items derived from posterior cord injury are included in the assessment items. We experienced a case in which a patient was diagnosed with an intradural extramedullary tumor in the thoracic spinal cord, and after surgery to remove the tumor compressed from behind the dura mater, he was transferred to the convalescent rehabilitation unit to recover from posterior cord injury due to the necessity of implementing rehabilitation. We report here a case report of a spinal cord disorder that improved the recovery process of posterior cord injury using the cFAS index because there is no case report of a spinal cord injury that improved the process using the cFAS index.

The cFAS assesses physical function and rehabilitation interventions and offers four advantages: ⅰ) it is useful in determining the effects of detailed rehabilitation; ⅱ) it facilitates improvements to rehabilitation programs based on assessment of effects (trajectory modification); ⅲ) it has the psychometric properties required in a rating scale; and ⅳ) it enables clinical study of rehabilitation medicine for cancer. Diseases covered by previous cFAS studies include gastrointestinal, pulmonary, cerebral, hematologic, urological, genital, and head and neck cancers [3]. Furthermore, cFAS endpoints include standing on one leg with eyes open and closed (also included as Functional Balance Scale (FBS) [4] items); this index was used because it can assess ataxia related to position sense disturbance and observe changes over time. American Spinal Injury Association [1,2] standards for neurological and functional classification of spinal injury utilize evaluation of muscle strength and superficial sensation, making it difficult to be used as an index of recovery from spinal cord injuries with deep sensory impairment derived from position sense disturbance.

Here we report the case of a patient who underwent surgical resection of an intradural extramedullary tumor of the thoracic spine (Th6) and was transferred to a convalescent rehabilitation ward with position sense disturbance due to posterior compression.

## Case presentation

This table is based on the Japanese version of the Cancer Functional Assessment Set (cFAS) [3] and is presented in this paper as the English version. The maximum value of this table is listed as 102 points and the minimum value as 0 points. The specific values of this case are listed in Table 1. 

**Table 1:** Cancer Functional Assessment Set (cFAS) Maximum 102 points, Minimum 0 points

Assessment parameters	Points	0	1	2	3	4	5
Getting up		Total assistance to maximal assistance	Moderate assistance	Minimal assistance	Supervision	Use of assistive devices	Complete independence
Standing up							
Getting on top							
Walking 50 m							
Going up and down stairs (1 flight)							
Grip strength (in the sitting position with the elbow extended)	Right	<10 Kg	≥10 but <15 Kg	≥15 but <20 Kg	≥20 but <25 Kg	≥25 but <30 Kg	≥30 Kg
	Left						
Muscle strength of the iliopsoas muscle	Right	MMT^*^0	MMT^*^1	MMT^*^2	MMT^*^3	MMT^*^4	MMT^*^5
	Left						
Muscle strength of the quadriceps muscle	Right						
	Left						
Muscle strength of the anterior tibial muscle	Right						
	Left						
Standing on one leg with eyes open	Right	Not feasible	1-2 seconds	3-4 seconds	5-6 seconds	7-9 seconds	≥10 seconds
	Left						
Standing on one leg with eyes closed (for 1 minute)		Not feasible	Trunk oscillation ≥10cm	Trunk oscillation ≥5 but <10cm	Trunk oscillation < 5cm		
Trunk muscle strength		Inability to get up from a 45° inclined sitting position	Able to get up from a 45° inclined sitting position in the absence of resistance	Able to get up from a 45° inclined sitting position with light resistance	Able to get up from a 45° inclined sitting position even with strong resistance		
Passive shoulder abduction range of motion	Right	<140°	≥140 but < 165°	≥165 but <175°	≥175		
	Left						
Passive ankle dorsiflexion range of motion (with knees flexed)	Right	<5°	≥5 but <15°	≥15 but < 25°	≥25		
	Left						
Upper limb sensory function	Left or Right	Severe disability	Moderate disability (issues with movement)	Mild disability (no issues with movement)	Normal		
Lower limb sensory function	Left or Right	Severe disability	Moderate disability (issues with movement)	Mild disability (no issues with movement)	Normal		
Major range of activity		On the bed	Within a room	Within one’s home/the ward	Within the hospital/outdoors		
Total points							

The patient was a 68-year-old male engaged in the printing business. In November 2017, the patient began to feel discomfort in the toes followed by lightheadedness starting in January 2018. He visited three local physicians’ offices in February 2018; however, the cause was not identified. Although magnetic resonance imaging of the lumbar spine was performed by a local physician in April 2018, abnormalities were not identified. Thereafter, the patient experienced difficulty in climbing stairs and onset of dysuria. In May 2018, muscle weakness in both lower limbs worsened and the patient experienced difficulty in walking. On May 8, the patient lost his balance and fell off his motorcycle. On May 10, the patient was referred to our institution’s neurology department and emergently hospitalized.

Manual muscle testing showed a decrease in muscle strength in both lower limbs as well as a decrease in deep and superficial sensations in the foot soles to the lower legs. An intradural extramedullary tumor in the Th6 region of the thoracic spine was identified via thoracic spine magnetic resonance imaging; the patient underwent orthopedic surgery, and tumor resection was performed on May 21. Pathological results led to the diagnosis of meningioma, and the patient was transferred to the convalescent rehabilitation ward of our institution on June 8, 18 days after surgery.

Ethics

Symptoms present and evaluations are described in accordance with the 2001 International Classification of Functioning Disability and Health from the World Health Organization [5]. The ICF is a global standard for conceptualizing and classifying functioning and disability, agreed upon by the World Health Assembly in 2001. It is an international frame of reference for information on health and disability and provides a freely available resource. We use this resource to describe case reports. We also obtained written informed consent from the patient to publish this case report and accompanying images, in accordance with the Declaration of Helsinki.

Mental and physical function/structure at admission

Consciousness was clear, with no dysarthria, and there were no abnormalities in the cranial nervous system. Motor system measures: grip strength in the right and left hands was 28 and 27 kg, respectively; manual muscle testing (right/left) of strength in the upper limbs was 5/5, trunk was 4, iliopsoas muscle was 3/3, quadriceps was 3/3, biceps femoral was 3/3, tibialis anterior was 2/2, and triceps were 3/3. Sensory system measures: superficial sensation was predominantly in the right lower limb as well as 8/10 hypoesthesia. Deep sensation: severe obtundation was observed predominantly on the right. Deep tendon reflex: patellar and Achilles tendon reflexes (1+) were confirmed. There was no dysfunction of the bladder and bowel. Positive results were obtained in the Romberg and Mann tests. The patient was unable to stand on one leg, and an ataxic gait was observed. American Spinal Injury Association Category was D. Basic functions included standing up to mobility/transfer and independence. Walking was feasible if the patient walked for 40 m with a walker under supervision. Self care: minimal assistance was required when going to the toilet and bathing; otherwise, the patient was independent. The FBS [4] and cFAS [3] scores were 18/56 and 55/102 points, respectively; Functional Independence Measure (FIM) [6] (motor components) 58 points + (cognitive components) 32 points = total 90 points.

Examinations

**Figure 1:**
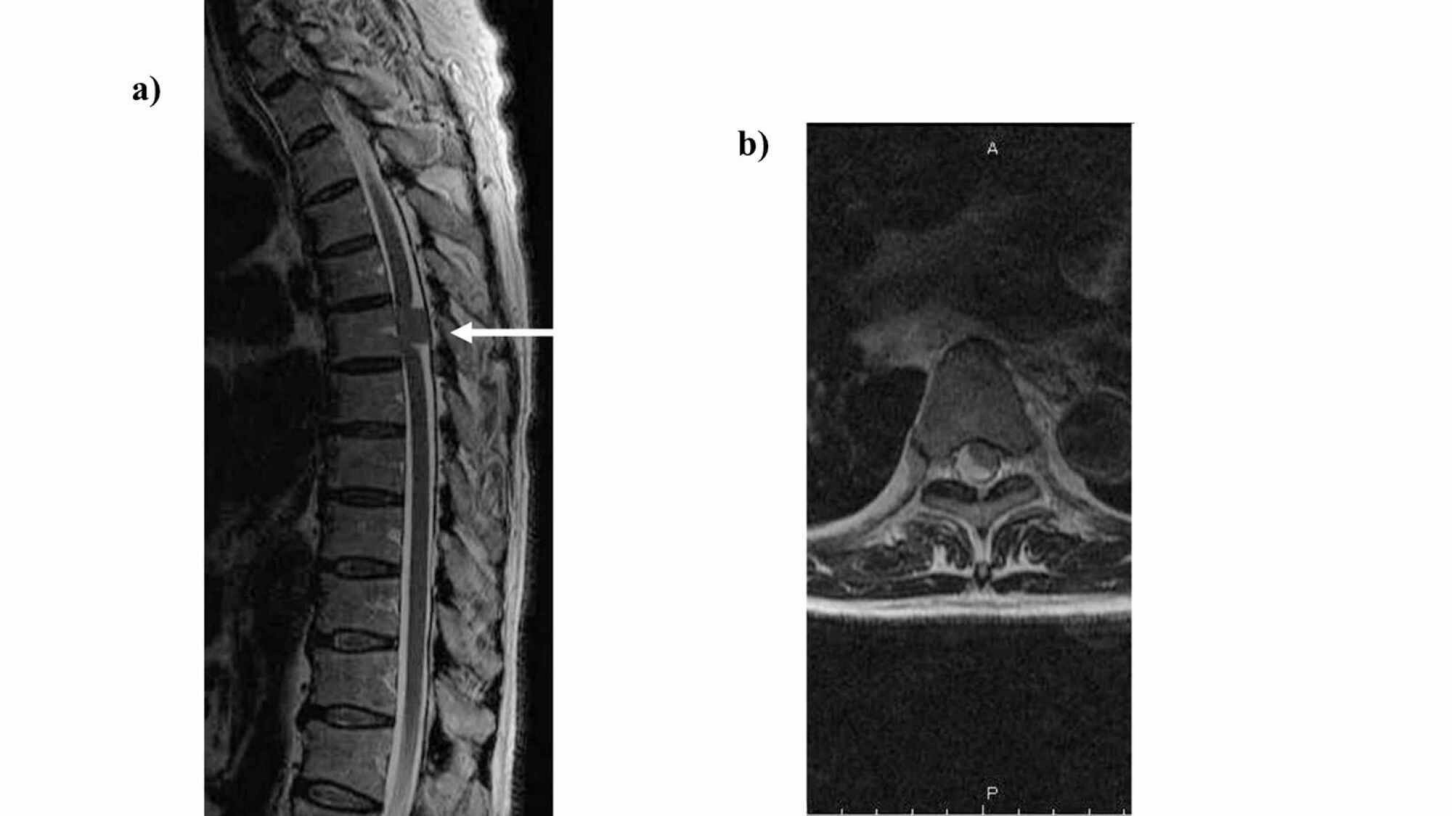
Thoracic spine intradural extramedullary tumor (Th6). Preoperative images. a) Sagittal plane of thoracic spine MRI: The arrows indicate the location of the thoracic spine intradural extramedullary tumor (Th6). It compresses spinal cord from posterior. b) Transverse of thoracic spine MRI: The slice containing the tumor in transverse section was not imaged and is the slice above it. The spinal cord is compressed by the tumor from behind, and the intramedullary nerves are deflected anteriorly.

**Figure 2:**
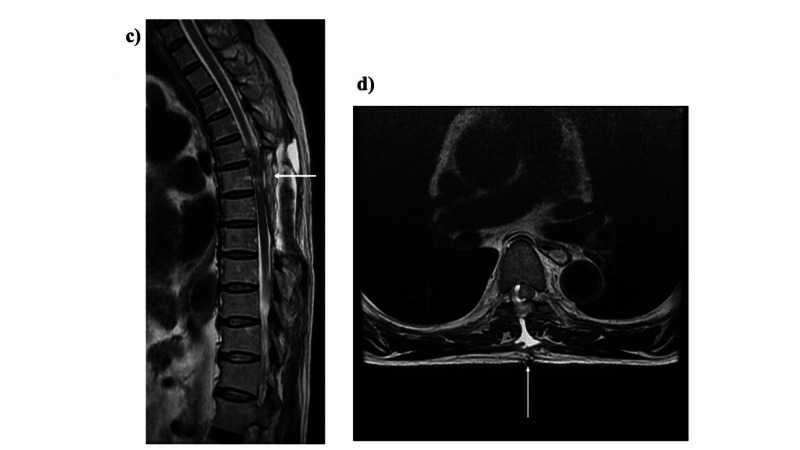
Thoracic spine intradural extramedullary tumor (Th6). Postoperative images. c) Sagittal plane of thoracic spine MRI: This image shows the removal of an extramedullary tumor, with the spinous processes in the Th5 to Th8 region removed. The arrows indicate where the tumor was removed. d) Transverse of thoracic spine MRI: the compressed tumor has been removed, but the spinal fluid was leaking.

Thoracic spinal cord magnetic resonance imaging was performed (Figure 1-2). Based on the aforementioned findings, the following four points were identified as issues: (ⅰ) ataxic gait derived from position sense disturbance due to posterior compression from the tumor, (ⅱ) requirement of a walker due to muscle weakness in both lower limbs, (ⅲ) minimal assistance needed for activities of daily living, and (ⅳ) difficulty returning to work. The following were proposed rehabilitation treatment plans: range of motion training, muscle strength enhancement training, outdoor walking, stair climbing, applied movement training, balance motion acquisition training, activities of daily living, housework, and standing walking under a weight load used to enable a return to work.

Intervention

Physical therapy was provided for gross motor function, ataxic gait improvement. Occupational therapy was provided for fine motor impairments or difficulties with activities of daily living. These therapies were provided for a total of 3 hours per day during hospitalization.

Outcomes

The patient initially had intense deep hypoesthesia in both lower limbs and decreased superficial sensation on the right, which evolved with ataxic gait derived from position sense disturbance due to posterior compression from the tumor. The FIM [6] (motor components), cFAS [3], and FBS [4] at the time of admission were 58, 55, and 18 points, respectively. Rehabilitation treatment was continued, the decrease in superficial sensation gradually disappeared, and only a slight decrease in deep sensation remained, along with resolution of the ataxic gait. At discharge, the FIM (motor components) was 88 points (admission + 30 points), cFAS was 86 points (admission + 31 points), and FBS was 52 points (admission + 34 points), showing improvement compared with those at admission (Table 2). The patient was discharged on July, 2018 and returned to his printing work in August 2018.

**Table 2:** Cancer Functional Assessment Set (cFAS), Functional Independence Measure (FIM), Functional Balance Scale (FBS) Scores at Admissions and Discharge cFAS: Two points were added for each item of getting up and going up and down stairs and each item of muscle strength, four points for standing on only the right leg with eyes open and two points for only the left leg with eyes open, three points for standing on one leg with eyes closed, and two points for the range of activity. FIM:  One point each was subtracted if handrails or canes were used to move to bathtubs, use stairs, or to walk.

	At the time of admission	At discharge	Comparison with results at the time of admission
FIM (motor components)	58	88	+30 points
cFAS	55	86	+31 points
FBS	18	52	+34 points

## Discussion

We described the case of a patient with an intradural extramedullary Th6 meningioma who underwent resection six months after the onset of disease and was able to return to work three months after surgery. Predominant postoperative symptoms were residual ataxic gait derived from position sense disturbance due to posterior compression from the tumor, which mainly presented as requirement of a walker to walk. The ataxic gait derived from the position sense disturbance gradually reduced with rehabilitation treatment, and the FIM and cFAS improved by 30 and 31 points, respectively, compared with those at admission. Immediately before being discharged from the hospital, the patient underwent applied exercise training and training for housework, such as moving around with a heavy load of around 10 kg, which led to the patient being discharged for home and returning to work. In terms of changes for each endpoint, the cFAS, FIM, and FBS improved by 31, 30, and 34 points, respectively, from admission to discharge. The FBS includes items with high difficulty, such as changing feet when stepping onto stairs, maintaining standing position with the switched foot, and maintaining standing on one leg; in addition, maintaining standing with eyes open and closed were included in the cFAS evaluation. Thus, the improvement in FBS may have led to the improvement in cFAS score. A retrospective study of 59 patients showed correlations between cFAS and FIM scores, suggesting utility index as the physical assessment of patients with cancer [7]. Although an assessment of correlation was not feasible in our patient, both evaluations showed improvement.

In a previous report of 97 patients with spinal intradural extramedullary tumors, tumors in the thoracic spine level accounted for 63% during all patients, and meningioma accounted for 38.1% for classification of tumor. Moreover, postoperative improvement tended to be faster in patients with good outcomes from initial onset to surgery, with postoperative improvement tending to be 4.58 ± 2.57 months in patients with good outcomes and 6.46 ± 5.99 months in those with poor outcomes [8]. The reported postoperative follow-up period in these patients ranged from three to 44 months, [9] whereas our patient was discharged 69 days after surgery and was able to return to work 72 days after surgery, showing a much faster course of improvement compared with that in the previous report. Thus, performing surgical resection within six months from the onset of subjective symptoms may have been a factor leading to the early improvement.

Among the International Statistical Classification of Functioning (ICF) components used as an international classification of functionality that was determined by the World Health Organization (WHO) in 2001, personal and environmental factors as well as participation were affected in our patient; specifically, climbing up and down stairs was necessary for our patient who lived alone, and use of a motorcycle for commute became an issue during the course. When the patient was asked to forego the motorcycle for the time being and use a train, he was able to return to work. With improved FBS, cFAS, and motor components of the FIM to 88 points (leading to a high total score of 123 points), reinforcement of on-floor movements and training for standing up from the floor were important factors that contributed to his ability to live at home.

Our case report contributes new knowledge regarding FBS, cFAS, and FIM assessments by providing information on functional sensory disturbance from posterior spinal cord. The improvements in cFAS and FIM scores over time with rehabilitation reflected improvements in activities of daily living during recovery from position sense disturbance. The cFAS can be an effective assessment for posterior compression of the spinal cord in patients with cancer.

Study limitations

This case report has several limitations. It could not be generalized because it involved a single case from a single facility and any correlations for/between each assessment could not be evaluated. Therefore, we recommend further research to identify the generalizability of criteria for evaluation in patients with posterior compression of the spinal cord.

## Conclusions

Our experience using the cFAS, FIM, and FIM in a patient with a thoracic spine intradural extramedullary tumor indicates that these indexes positively contribute to the assessment index of physical function and rehabilitation interventions in patients with cancer. However, considering the limitations of this case report, no conclusions can be made regarding causality between these indices and the patient’s functional improvements. Therefore, further research to identify the generalizability of criteria for evaluation in patients with posterior compression of the spinal cord is recommended.
